# Association of self-reported hearing loss and tinnitus with brain structure and cognitive function: a population-based cross-sectional analysis from the Study of Health in Pomerania (SHIP)

**DOI:** 10.1186/s13195-026-02166-6

**Published:** 2026-08-27

**Authors:** Claudia Schwarz, Stefan Frenzel, Till Ittermann, Ulrike Grittner, Silke M Wortha, Tina Brzoska, Hans J Grabe, Agnes Flöel, Friedrich Ihler

**Affiliations:** 1 Department of Neurology, University Medicine Greifswald, Greifswald, Germany; 2 Department of Psychiatry, University Medicine Greifswald, Greifswald, Germany; 3 Institute for Community Medicine, University Medicine Greifswald, Greifswald, Germany; 4 Institute of Biometry and Clinical Epidemiology, Charité – Universitätsmedizin Berlin, Berlin, Germany; 5 Berlin Institute of Health (BIH), Berlin, Germany; 6 Centre for Mathematical Cognition, Loughborough University, Loughborough, UK; 7 Department of Otorhinolaryngology, Head and Neck Surgery, Greifswald University Medicine, Greifswald, Germany; 8 German Center for Neurodegenerative Diseases (DZNE), Standort Rostock/Greifswald, Greifswald, Germany

**Keywords:** Hearing loss, Tinnitus, Epidemiology, Dementia prevention, Alzheimer’s disease, Magnetic resonance imaging, Risk factors, Memory

## Abstract

**Background:**

Hearing loss has been reported as a major, potentially modifiable risk factor for dementia and often co-occurs with tinnitus. Both conditions are prevalent and have been linked to brain health and cognitive decline. However, evidence from studies containing the whole age span of adults remains limited. Therefore, this study investigated associations of hearing loss and tinnitus with brain structure and cognitive performance in a large population-based cohort from northeastern Germany.

**Methods:**

Data from the baseline investigation of 4,420 participants (age 20–84 years) for the SHIP-TREND cohort was analysed. Thereof, 4,400 completed a self-assessment for hearing and 3,937 for tinnitus. Cognitive performance was assessed by tests on verbal memory and executive function. T1-weighted magnetic resonance imaging yielded data on global and regional volumes and cortical thickness for 2,144 participants. Multiple linear regression and mediation analyses were conducted, adjusted for age, sex, and further risk factors for dementia.

**Results:**

A total of 1,455 participants reported hearing loss, 679 thereof a clinically relevant degree while 874 participants reported tinnitus. Hearing loss was associated with worse memory performance and unfavourable brain measures, particularly along the central auditory pathway and in regions implicated in cognitive function and Alzheimer’s Disease. Notably, white matter hypointensity volume partially mediated (8.8%) the association of self-reported hearing loss with immediate recall performance. Self-reported hearing loss was associated with tinnitus (odds ratio 7.5 [95%CI 6.0 to 9.3]; *p* < 0.001), but no relevant combined effects on cognitive performance or brain measures were observed, and tinnitus alone was not associated with cognition or adverse brain measures.

**Conclusions:**

This large cohort study not only confirmed independent associations of self-reported hearing loss with memory performance and structural brain measures but also suggested a pathophysiologic link between hearing loss and cerebrovascular disease. Our findings support an association between hearing loss, but not tinnitus, and cognitive decline as well as dementia-related brain changes, highlighting hearing loss as a potentially important target for early prevention strategies.

**Supplementary Information:**

The online version contains supplementary material available at https://doi.org/10.1186/s13195-026-02166-6.

## Background

Hearing loss is a highly prevalent condition, with moderate or greater severity affecting approximately 5% of the global population [1]. It has a close but not yet fully understood association with tinnitus, another form of auditory dysfunction affecting up to 14% of adults worldwide [2]. Hearing impairment seriously affects multiple domains of daily functioning, including quality of life and social participation, and has been associated with an increased risk of cognitive decline and dementia with advancing age. The 2024 *Lancet* Commission report identified 14 potentially modifiable risk factors that could prevent or delay nearly half of all dementia cases, including midlife hearing loss as a comparably prominent factor contributing 7% [3].

Previous studies have linked hearing loss to impaired cognitive performance, faster cognitive decline, and increased dementia risk [3–8]; with similar associations reported for tinnitus [9]. Furthermore, hearing loss and tinnitus have also been associated with distinct structural brain changes in older adults, including atrophy and compensatory neuroplasticity [6, 10–13]. In detail, hearing loss has been linked to grey matter (GM) atrophy in temporal regions including the auditory cortex, hippocampus, and entorhinal cortex - regions vulnerable to Alzheimer’s Disease (AD) [6, 11–13]. Further evidence exists for an association of hearing loss with global and regional white matter (WM) degradation, especially in cognitive processing regions including the temporal lobe and hippocampus [14, 15]. In contrast, tinnitus has been associated with larger hippocampal and thalamic volumes [11]. Previous studies reported associations between hearing impairment, cognition, and brain structure, with patterns varying by laterality, duration, and severity of hearing impairment.

Several mechanisms have been proposed to explain these associations, including increased cognitive load from reduced auditory input, auditory deprivation leading to brain atrophy, shared cardiovascular or neuronal pathology simultaneously affecting cochlear and brain tissue, and social isolation and depression as downstream consequences of hearing loss [10, 12, 16]. Ageing may be a shared determinant, with age-related hearing loss reflecting neuronal and metabolic deterioration across the lifespan [2, 17].

Hearing loss has been identified as one of the major modifiable risk factors for dementia that often remains untreated, but its association with cognition, brain structure, and tinnitus has not yet been fully elucidated. Therefore, this study aims to further explore these associations in a cross-sectional analysis from a large population-based cohort, investigating baseline data on self-reported hearing loss and tinnitus, cognitive performance of different domains as well as brain structure, including GM and WM volumes, cortical thickness, and WM hypointensity (WMH) volume derived from T1-weighted magnetic resonance imaging (MRI). We tested for associations and possible mediations, while adjusting for evidence-based risk factors for cognitive decline and dementia.

## Methods

### Study design and participants

The Study of Health in Pomerania (SHIP) is a population-based study conducted at University Medicine Greifswald, currently comprising three cohorts and designed to investigate risk factors, subclinical disorders, and clinical diseases [18]. For the present analysis, we used baseline data (SHIP-TREND-0, 2008–2012) from the SHIP-TREND cohort, with a total of 4,420 participants. Data collection included self-completed questionnaires, structured interviews, comprehensive interdisciplinary assessments, whole-body MRI, and biosampling. As it was restricted to data from the baseline investigation, this analysis is cross-sectional. Further details on study design and participants are provided in the appendix (eMethods 1 in Supplement 1).

### Cognitive outcomes

Cognitive performance was assessed using the wordlist task from the Nuremberg Gerontopsychological Inventory [19] and the Stroop test [20]. The wordlist was employed to assess free reproduction and delayed recognition of verbal memory. In the first part of the test (free reproduction), eight words are read to the participant. Immediately afterwards, the participant is asked to repeat as many words as possible. The number of correctly reproduced words is recorded (immediate recall score; range 0 to 8). Thirty minutes later, the delayed recognition test is performed. Sixteen words are read to the participant, eight of which are the same as in the first test and eight new words are added. The participant is asked to answer “yes” or “no” to indicate whether the word has been used before or not. The recognition test is evaluated by subtracting the incorrect “yes” answers from the correct “yes” answers (delayed recognition score; range − 8 to 8). The subscores of the free reproduction and the recognition test are added together to obtain the total score of the wordlist (total score; range − 8 to 16).

The Stroop test assesses executive function, particularly selective attention and inhibitory control. The test consists of three conditions: (1) reading colour words printed in black ink (colour-word-naming) (2), naming the colours of coloured boxes (colour-box-naming), and (3) naming the ink colour of incongruently printed colour words while suppressing the automatic reading response (incongruent condition). Performance was assessed based on the time required to complete each condition. As a value for selective attention, we calculated the Stroop interference score, following the Stroop paradigm originally introduced by Stroop [20] and consistent with previous analyses of the SHIP dataset [21]. This score was defined as the difference in completion time between the incongruent and the colour-box-naming conditions. A lower interference score suggests better ability to overcome this interference, indicating stronger cognitive control and executive function. After excluding participants with incomplete data, problems during testing, and unrealistic values for colour-word-naming (< 1s), colour-box-naming (< 15s), or incongruent conditions (< 21s), a total of 4,025 participants remained for our analysis.

### Brain imaging outcomes

Brain imaging data were collected on a Siemens 1.5 T MRI scanner (Siemens Magnetom Avanto scanner; Siemens, Erlangen, Germany) at University Medicine Greifswald, Germany, for a randomly selected subgroup of SHIP-TREND-0 participants (*N* = 2,144). T1-weighted images were processed using FreeSurfer v7.4 (http://surfer.nmr.mgh.harvard.edu/) for cortical reconstruction and volumetric segmentation (eMethods 2 in Supplement 1). Analyses included global and regional brain volume and cortical thickness measures. Global measures comprised total, cortical, cerebellar and subcortical GM and WM volumes, mean cortical thickness, and WMH volume. Regions of interest (ROIs) were selected based on their position along the auditory pathway and established links to cognition and dementia [12], including volumes of the brainstem, thalamus, and hippocampus, as well as volume and cortical thickness of the superior temporal gyrus. All values were averaged across hemispheres. Estimated total intracranial volume (eTIV) was included as a covariate in volumetric analyses to adjust for head size.

### Primary exposure and risk factors

Exposure variables included self-reported hearing loss and tinnitus from standardised written questionnaires. The respective questions were part of two separate lists assessing a variety of symptoms. For both self-reported hearing loss and tinnitus, introductory phrases asked to consider each symptom separately and grade it according to severity using Likert-type response scales. Options ranged from no occurrence to “severely” (hearing loss) or “always” (tinnitus). For the questions, hearing loss was referred to by two different German terms covering both the specific medical condition (“Schwerhörigkeit”) as well as a broad term for any hearing difficulties in general (“Hörbeschwerden”). Tinnitus was assessed by initially asking if the symptom occurred at all. A follow-up question then allowed for further grading if the first answer was positive. The original German wording of the questions and the response options at each grade including a translation are given in Table 1.

**Table 1:** Phrasing and grading of self-reported hearing loss and tinnitus

	Original questions *English translation*	Original response options *English translation*			
Introductory remarks for symptom list that included hearing loss.	Im Folgenden wird eine Reihe von Beschwerden genannt. Machen Sie bitte bei jeder aufgeführten Beschwerde ein Kreuz in eines der vier Kästchen, je nachdem, ob sie gar nicht, kaum, mäßig oder stark unter diesen Beschwerden leiden. In the following, several symptoms will be named. Please give an answer to every below-mentioned symptom and decide where to tick depending on how strong you suffer from these symptoms.				
Hearing loss occurrence and severity	Schwerhörigkeit, Hörbeschwerden *Hearing loss*,* hearing difficulties*	Gar nicht *Not at all*	Kaum *Rarely*	Mäßig *Moderately*	Stark *Severely*
Introductory remarks for symptom list that included tinnitus.	Leiden Sie an den im folgenden genannten Beschwerden? *Do you suffer from one of the following symptoms?*				
Tinnitus occurrence	Ohrgeräusche, Ohrensausen *Tinnitus/ ringing in the ears*	Nein *No*	Ja *Yes*		
Tinnitus severity	Wenn ja, wie häufig? *If yes*,* how often?*		Manchmal *Sometimes*	Häufig *Frequently*	Immer *Always*
Severity grades		0	1	2	3

We further included possible confounders into our statistical models, including age and sex as well as other currently assumed risk factors for dementia and cognitive decline The recent report of the *Lancet* commission on dementia prevention identified 14 modifiable risk factors that may account for nearly half of dementia cases worldwide [3]. Hearing loss is among these factors, and we were able to include eleven additional risk factors as covariates into our models. These included years of education, cardiovascular risk factors operationalised as metabolic syndrome (encompassing diabetes, hypertension and obesity/body mass index), depression, physical activity, smoking, alcohol consumption, social isolation, and vision. No data on air pollution was available in SHIP-TREND-0. Furthermore, traumatic brain injury could not be included due to substantial missing data, as the relevant variable (head trauma as cause of accident) was missing in 2,395 participants.

In detail, age at baseline examination was used in the analysis. Sex was documented in binary categories. Education years were coded as the standard duration of schooling associated with the highest completed school-leaving or vocational qualification rather than the actual number of years spent in education. Participants who were still attending school or who had not obtained a formal school or vocational qualification at the time of assessment were coded as having zero years of education. Smoking status was categorised as never, former or current smoker. Alcohol consumption over the last 30 days was calculated in grams of ethanol per day using a quantity–frequency questionnaire [22]. Physical activity was assessed during the interview and categorised as <1 h leisure or ≥1 h of physical activity per week. Metabolic syndrome was defined according to a modified version of the criteria by Alberti and colleagues [23], requiring the presence of at least three of five parameters: (i) present diabetes mellitus (defined by ≥ 8mmol/L non-fasting random blood glucose, ≥ 6.1 mmol/L fasting random blood glucose, or antidiabetic therapy), (ii) abdominal obesity (waist circumference ≥ 94 cm in men or ≥ 80 cm in women), (iii) low HDL-cholesterol (< 1.03 mmol/L in men or < 1.29 mmol/L in women), (iv) elevated triglycerides (≥ 2.3 mmol/L non-fasting or ≥ 1.7 mmol/L fasting triglycerides, or lipid-lowering medication), (v) present hypertension (≥ 130/85mmHg or antihypertensive therapy) [24]. Depression was assessed using a self-report questionnaire based on three items of the Composite International Diagnostic – Screener (CID-S) and categorised as binary [25, 26]. Social isolation was captured by two interview questions on marital status and living conditions of the participants and categorised into four levels. Vision was assessed via a self-report using a six-level scale ranging from excellent to completely blind.

### Statistical analysis

Demographics and characteristics of the participants as well as the quality of hearing and tinnitus in our study cohort were analysed descriptively using means, standard deviations, percentages, and ranges for the overall cohort as well as stratified by levels of self-reported hearing loss. Multiple linear regression models were conducted to analyse the association between self-reported hearing loss (independent variable) and cognitive performance (dependent variable) as well as MRI brain measures (dependent variable). Mediation analyses were performed using the R package “mediate” [27] to evaluate whether brain measures mediate the direct association between self-reported hearing loss and cognitive performance. For implementing the mediation models, the independent variable on self-reported hearing loss had to be transformed into binary categories (dichotomised variable), combining grade 0 (“not at all”) and grade 1 (“rarely”) for no substantial self-reported hearing loss and grade 2 (“moderately”) and grade 3 (“severely”) for moderate and severe self-reported hearing loss [28]. Moreover, the covariate vision had to be transformed from a categorical into a numerical variable due to the very low sample sizes in some categories. Metric variables were standardised prior to analysis, whereas the binary independent variable on self-reported hearing loss and several factor covariates were left unstandardised. We report partially standardised regression coefficients and p-values for the direct and indirect pathway effects as well as their 95% confidence intervals (CIs), estimated via bootstrapping with 10,000 simulations each.

Moreover, we evaluated the interaction of self-reported hearing loss and tinnitus and its associations with cognitive function and brain measures. First, associations between self-reported hearing loss and tinnitus were analysed using Fisher’s exact test. Second, a new factor variable with four levels (neither hearing loss nor tinnitus, tinnitus but no hearing loss, hearing loss but no tinnitus, both hearing loss and tinnitus) was computed and included in the regression models as an independent variable of interest. All models were adjusted for potential confounders as described above. In addition, eTIV was included as a covariate in all models investigating volumetric brain measures. In all analyses, participants with missing data on one of the variables included in the model were excluded from the respective analysis. For the mediation analyses, baseline characteristics were similar between participants included in the analyses and those excluded due to incomplete data (eTable 1 in Supplement 1). In addition, in subgroup analyses among middle-aged and older adults (midlife: 18–60 years; late life: >60 years) we examined the associations between self-reported hearing loss (independent variable) and cognitive performance (dependent variable) as well as MRI brain measures (dependent variable) using multiple linear regression models.

Statistical analyses were performed using R version 4.4.1. All tests were two-sided and no adjustments for multiple testing were performed. Because formal statistical significance is only relevant for a priori primary hypotheses, we interpreted p-values cautiously and considered effect estimates to gauge clinical relevance. Further details on statistical analysis are provided in the appendix (eMethods 3 in Supplement 1).

## Results

### Characteristics of study participants

In total, 4,420 individuals underwent the cross-sectional baseline investigation of the SHIP-TREND cohort (SHIP-TREND-0) and were included in the present analysis. Thereof, 4,400 (99.5%) completed the structured self-assessment for hearing, with 1,455 individuals (33.1%) reporting any hearing loss wherefrom a clinically relevant hearing loss, i.e. at least moderate, was found for 697 (15.8%) individuals. In addition, 3,937 (89.1%) individuals completed the self-assessment for tinnitus, with 874 (22.2%) individuals reporting any tinnitus. Table 2 gives an overview of the characteristics of the cohort as well as the frequency and severity of self-reported hearing loss and tinnitus. Moreover, participant characteristics, including sex and years of education, were largely comparable across levels of self-reported hearing loss (eTable 2 in Supplement 1). However, participants with more severe self-reported hearing loss were older than those reporting rare or no hearing loss. Further details on participants’ characteristics are provided in eTables 3 and 4 in Supplement 1.

**Table 2:** Characteristics of study participants

Characteristic	Total sample (*N* = 4,420)
Females [N] (%)	2,275 (51)
Age [years]	52 (15), 20–84
Education [years]	12 (3), 0–17
Self-reported hearing loss (*N* = 4,400) [N] (%)	
not at all	2,945 (66.9)
rarely	758 (17.2)
moderately	541 (12.3)
severely	156 (3.5)
Self-reported tinnitus (*N* = 3,937) [N] (%)	
no	3,063 (77.8)
sometimes	436 (11.1)
frequently	160 (4.1)
always	277 (7.0)
unsure	1 (< 0.1)

Data are given as mean (standard deviation) and range or number (%). Missing data for self-reported hearing loss (*N* = 20) and tinnitus (*N* = 483) are reported

### Self-reported hearing loss and cognitive function

We observed associations between self-reported hearing loss and cognitive function (Table 3). Participants with self-reported moderate hearing loss showed worse memory performance in the total score of the wordlist (unstandardised regression coefficient (*B*) -0.34 [95% CI -0.59 to -0.10]; *p* = 0.006) as well as in the immediate recall score (*B* -0.23 [95% CI -0.35 to -0.11]; *p* < 0.001) compared to participants with no hearing loss. Participants with self-reported severe hearing loss demonstrated worse cognitive function in all sub scores of the wordlist, the total score (*B* -0.93 [95% CI -1.35 to -0.50]; *p* < 0.001), immediate recall score (*B* -0.62 [95% CI -0.83 to -0.41]; *p* < 0.001) and delayed recognition score (*B* -0.31 [95% CI -0.60 to -0.02]; *p* = 0.038) compared to participants with no hearing loss. No associations were found between self-reported moderate hearing loss, delayed recognition score, and Stroop interference score, between self-reported severe hearing loss and Stroop interference score as well as between self-reported mild hearing loss and cognitive performance in all tested domains.

**Table 3:** Association of self-reported moderate and severe hearing loss with cognitive function

	Estimate	95% CI	t-value	*p*-value
Self-reported moderate hearing loss				
Wordlist, total [score]	-0.34	-0.59 to -0.10	-2.76	0.006
Wordlist, immediate recall [score]	-0.23	-0.35 to -0.11	-3.70	< 0.001
Wordlist, delayed recognition [score]	-0.11	-0.28 to 0.05	-1.35	0.176
Stroop interference score [s]	-0.22	-1.31 to 0.87	-0.39	0.698
Self-reported severe hearing loss				
Wordlist, total [score]	-0.93	-1.35 to -0.50	-4.25	< 0.001
Wordlist, immediate recall [score]	-0.62	-0.83 to -0.41	-5.69	< 0.001
Wordlist, delayed recognition [score]	-0.31	-0.60 to -0.02	-2.08	0.038
Stroop interference score [s]	1.45	-0.52 to 3.42	1.44	0.150

Linear models on the association between self-reported hearing loss and cognitive performance (wordlist, *N* = 3,978; Stroop test, *N* = 3,653), adjusted for age, sex, education, metabolic syndrome, depression, physical activity, smoking, alcohol consumption, social isolation, and vision

*Abbreviations*: *CI* Confidence interval, *s* seconds

When stratifying for age, the associations of self-reported moderate and severe hearing loss with cognitive performance across midlife and late life were largely consistent with those in the full sample (eTables 5 in Supplement 1). Particularly, self-reported moderate and severe hearing loss were associated with immediate recall performance across both age groups, and with the total score in midlife participants. No associations with delayed recognition performance were observed in either age group.

### Self-reported hearing loss and structural brain measures

We further observed associations between self-reported hearing loss and brain measures (Table 4). In detail, we found that participants with self-reported severe hearing loss showed smaller cerebral WM (*B* -10.48 [95% CI -19.44 to -1.53]; *p* = 0.022) and subcortical GM volume (*B* -0.99 [95% CI -1.81 to -0.177]; *p* = 0.017), including smaller regional volume of the brain stem, thalamus and hippocampus (all p’s < 0.034), as well as smaller mean cortical thickness (*B* -0.04 [95% CI -0.07 to -0.02]; *p* = 0.002) and superior temporal gyrus cortical thickness (*B* -0.06 [95% CI -0.10 to -0.03]; *p* = 0.001) compared to participants with no hearing loss. In addition, participants with self-reported severe hearing loss demonstrated greater WMH volume (*B* 0.82 [95% CI 0.17 to 1.46]; *p* = 0.013) compared to participants with no hearing loss. No associations with brain measures were observed for participants with self-reported moderate or rare hearing loss.

**Table 4:** Association of self-reported severe hearing loss with brain measures

	Estimate	95% CI	t-value	*p*-value
Global measures				
total GM volume [cm^3^]	-7.26	-14.52 to 0.01	-1.96	0.050
cortical GM volume [cm^3^]	-6.36	-12.80 to 87.45	-1.94	0.053
subcortical GM volume [cm^3^]	-0.99	-1.81 to -0.18	-2.38	0.017
cerebellar GM volume [cm^3^]	0.03	-2.48 to 2.55	0.03	0.978
cerebral WM volume [cm^3^]	-10.48	19.44 to -1.53	-2.30	0.022
cerebellar WM volume [cm^3^]	-0.31	-1.30 to 0.67	-0.62	0.536
WMH volume [cm^3^]	0.82	0.17 to 1.46	2.49	0.013
mean cortical thickness [mm]	-0.04	-0.07 to -0.02	-3.12	0.002
Regional measures				
brain stem volume [cm^3^]	-0.59	-1.08 to -0.10	-2.38	0.018
thalamus volume [cm^3^]	-0.33	-0.63 to -0.03	-2.13	0.033
hippocampus volume [cm^3^]	-0.25	-0.42 to -0.08	-2.94	0.003
superior temporal gyrus volume [cm^3^]	-0.42	-0.99 to 0.15	-1.44	0.149
superior temporal gyrus cortical thickness [mm]	-0.06	-0.10 to -0.03	-3.22	0.001

Linear models on the association between self-reported hearing loss and brain measures (*N* = 2,065), adjusted for age, sex, education, metabolic syndrome, depression, physical activity, smoking, alcohol consumption, social isolation, and vision. Models including volume measures as the dependent variable were further adjusted for estimated total intracranial volume

*Abbreviations*: *CI* Confidence interval, *GM* Grey matter, *WM* White matter, *WMH* White matter hypointensity

When stratifying for age, the associations of self-reported moderate and severe hearing loss with brain measures across midlife and late life were largely consistent with those in the full sample (eTables 6 in Supplement 1). Particularly, self-reported moderate and severe hearing loss were associated with immediate recall performance across both age groups, and with the total score in midlife participants. No associations with delayed recognition performance were observed in either age group.

### Mediation analyses

**Fig. 1:**
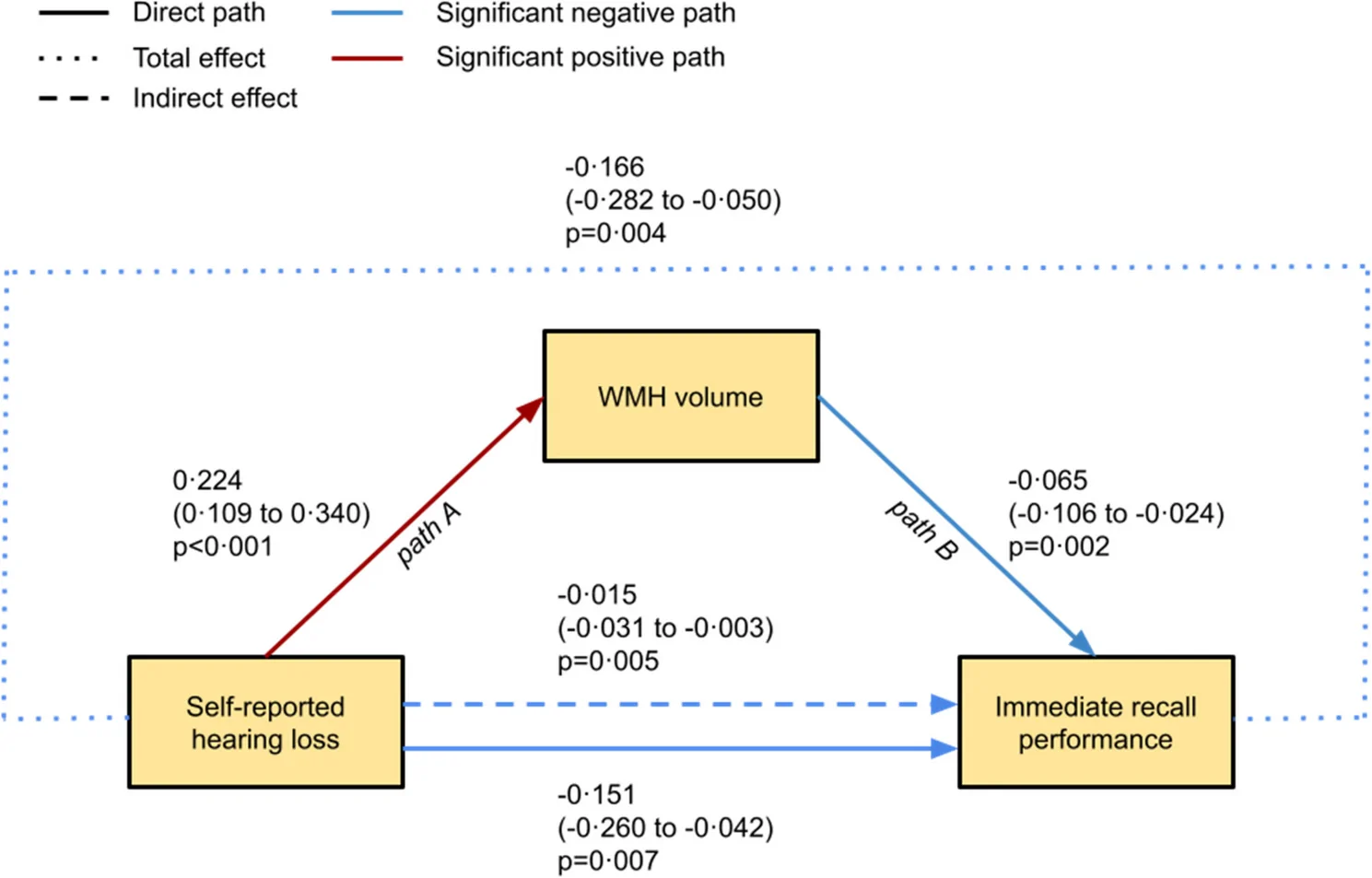
Mediating role of WMH volume on the relationship between self-reported hearing loss and memory performance. The path diagram of the mediation model included self-reported moderate and severe hearing loss as independent variable (X), immediate recall performance as dependent variable (Y) and white matter hypointensity (WMH) volume as mediator (M) (*N* = 2,050). The partially standardised results of the model for the direct path, indirect effect and total effect as well as the results for path A (X-> M) and path B (M-> Y) are provided, adjusted for age, sex, education, metabolic syndrome, depression, physical activity, smoking, alcohol consumption, social isolation, vision, and estimated total intracranial volume

We next assessed whether brain structure mediated the relationship between self-reported hearing loss and cognitive function. Mediation analyses were restricted to robust associations identified in the primary analyses between self-reported hearing loss and memory performance as well as brain measures. Thus, we tested the mediating effect of several pre-identified brain measures on the relationship of self-reported hearing loss and immediate recall performance in the wordlist. A complementary mediation effect was observed for self-reported moderate and severe hearing loss on immediate recall performance, with WMH volume acting as a mediator (Fig. 1). While there was an indirect effect of self-reported moderate and severe hearing loss on immediate recall performance via WMH volume (ab= -0.015 [95% CI -0.031 to -0.003]; *p* = 0.005), we also noticed the presence of a direct effect of self-reported moderate and severe hearing loss on immediate recall performance in the presence of the mediator (-0.151 [95% CI -0.260 to -0.042]; *p* = 0.007). WMH volume mediated 8.8% (proportion mediated: 0.088) of the total effect of self-reported moderate and severe hearing loss on immediate recall performance. For all other tested mediation models, no substantial effects were observed (eTable 7 in Supplement 1).

### Self-reported hearing loss and tinnitus

In our sample, 498 participants had both self-reported hearing loss and tinnitus, 779 had self-reported hearing loss only, 373 had tinnitus only, and 2,273 had neither. We observed an association between self-reported hearing loss and tinnitus (odds ratio 7.5 [95% CI 6.0 to 9.3]; *p* < 0.001). Participants with both self-reported hearing loss and tinnitus showed worse immediate recall performance (*B* -0.223 [95% CI -0.369 to -0.076]; *p* = 0.003) but better Stroop interference scores (*B* -1.423 [95% CI -2.762 to -0.084]; *p* = 0.037) compared with participants with neither condition. Tinnitus alone was associated with greater thalamic (*B* 141.7 [95% CI 6.2 to 277.3]; *p* = 0.041) and cerebellar GM volume (*B* 1235 [95% CI 108.1 to 2361.5]; *p* = 0.032). Further details are provided in the appendix (eResults 1, eTables 8 and 9, and eFigure 1 in Supplement 1).

## Discussion

In this large population-based study, self-reported hearing loss and tinnitus were highly prevalent and frequently co-occurred. Self-reported hearing loss was independently associated with worse memory performance and with structural brain alterations, particularly along the central auditory pathway and in regions implicated in cognitive function and AD. The association with memory was partly mediated by WMH volume, suggesting a potential link with cerebrovascular pathology. These findings were robust to adjustment for a wide range of established risk factors for cognitive decline and dementia. Although self-reported hearing loss and tinnitus were strongly associated, their co-occurrence was not related to additional differences in memory performance or brain structure. Tinnitus alone showed no associations with cognitive performance or adverse brain changes, but rather with larger brain volumes in specific regions.

Self-reported moderate or severe hearing loss was associated with lower memory performance, with the strongest associations for self-reported severe hearing loss and immediate recall as well as total wordlist score, and weaker associations for delayed recognition. Self-reported hearing loss was associated with 0.2 to 0.9 fewer words remembered, consistent with findings from previous population-based studies linking hearing loss to worse cognitive function across domains [4–8, 29], although we did not observe an association with executive function as assessed by the Stroop interference score. In contrast, Harrison Bush and colleagues [29] reported associations between hearing loss, assessed using pure-tone audiometry, and Stroop performance; but these were attenuated after adjustment for established risk factors. Differences in the methodological approaches used to calculate the Stroop interference score should also be considered [30], as different scoring methods may capture different levels or aspects of executive control and may therefore limit direct comparability between studies. Moreover, episodic memory is known to be affected early in ageing and in the course of AD, which may explain why associations were limited to this domain in our largely healthy cohort. In addition, associations were stronger for immediate recall than for delayed recognition performance, which may suggest that self-reported hearing loss has a greater impact on initial encoding than on recognition memory, a process that relies more heavily on retrieval. However, these findings should be interpreted in light of the task design, as the recognition task involved re-presentation of the target words, which may have reduced its cognitive demands relative to the immediate recall task. Moreover, although immediate recall is commonly used as a measure of episodic memory, performance on this task also depends on attentional processes, information span, and working memory capacity [31, 32]. Therefore, the observed associations between self-reported hearing loss and immediate recall may reflect broader effects on cognitive processes involved in information encoding and short-term retention. Furthermore, working memory plays an important role in speech understanding, particularly under adverse listening conditions [33]. Consistent with this framework, recent evidence suggests that working memory capacity may predict speech recognition under degraded listening conditions more strongly than age or peripheral hearing sensitivity [34]. These findings underscore the challenge of disentangling episodic memory and working memory contributions to immediate recall performance in the context of hearing loss. Furthermore, hearing loss has previously been linked to intermediary factors such as depression and social isolation, both established risk factors for cognitive decline and dementia [3, 16]. Thus, by controlling for those psycho-social risk factors, as well as for further currently assumed risk factors for dementia and cognitive decline, our findings support an independent association of self-reported hearing loss and verbal memory performance.

Self-reported severe hearing loss was associated with global and regional volumetric brain measures, including subcortical GM, brain stem, thalamus, hippocampus, cerebral WM, and WMH volume as well as mean and superior temporal gyrus cortical thickness. These results are consistent with earlier reports linking hearing loss with structural brain measures of several GM and WM regions, both in cross-sectional [8, 11, 12, 15] and longitudinal [6, 12–14] studies. A previous large-scale study including cohorts from three different geographic regions showed reduced volumes in widespread subcortical and temporo-parietal regions as well as lower integrity of WM tracts in participants with poorer hearing performance [8]. In the longitudinal, population-based Rotterdam Study applying pure-tone threshold audiometry, Boons and colleagues observed impaired WM microstructure associated with hearing loss while location and dynamic depended on the frequency profile of hearing loss [14]. Of particular interest in our findings were smaller GM volumes along parts of the central auditory pathway, including brain stem and thalamus, alongside lower cortical thickness in the superior temporal gyrus, supporting previous studies [8, 11–13]. These findings might be in line with proposed underlying mechanisms, including the sensory deprivation hypothesis, suggesting that auditory deprivation caused by hearing impairment leads to brain atrophy in brain structures important for cognitive function, or common causes, such as microvascular disease, affecting both hearing and cognition [10, 12]. Overall, self-reported severe hearing loss was associated with structural brain measures in regions linked to auditory processing, cognitive function, and AD development.

Mediation analysis showed that WMH volume partly mediated the association between self-reported hearing loss and immediate recall performance. This finding is consistent with the study of Wang and colleagues who also observed partly mediating effects of several structural brain measures (GM volume, WM mean diffusivity, and fractional anisotropy) on cognitive performance [8]. WMHs are common in the ageing brain and indicate pathological changes, often related to cerebrovascular disease and vascular risk factors, that are linked to early cognitive decline and AD pathology [35]. Although WM changes have been consistently linked to cognitive performance in healthy older individuals and within the AD continuum, evidence on its association with hearing loss per se remains limited [8, 12, 14]. Sudden sensorineural hearing loss (SSNHL), a distinct clinical subtype, stands apart in this regard. Although no increased WMH prevalence has been reported to date, individuals with SSNHL demonstrate cardiovascular risk factors more frequently and an increased risk of stroke has been described, suggesting an underlying cardiovascular pathomechanism [36]. Overall, our mediation analyses provide hypothesis-generating evidence that cerebrovascular disease may be involved in the observed association between hearing loss and cognition; however, this hypothesis requires confirmation in longitudinal studies.

Self-reported hearing loss was strongly associated with tinnitus, consistent with previous reports [2]. However, their co-occurrence did not lead to additive effects on memory performance or brain structure. This finding aligns with evidence suggesting that hearing loss and tinnitus are linked to distinct patterns of structural brain alterations. The presence of tinnitus has been associated with GM changes alongside specific, potentially compensatory, neuroplastic changes [11, 37, 38]. In line, our evaluation of participants with tinnitus but no hearing loss revealed greater volume of the thalamus and greater cerebellar GM compared to individuals with neither hearing loss nor tinnitus. In addition, tinnitus alone was not associated with cognitive performance. Furthermore, participants with both self-reported hearing loss and tinnitus showed better Stroop interference scores than participants with neither condition, contrary to previous findings [39, 40]. This inconsistency with previous studies might partly be attributable to differences in the methodological approaches to calculate the interference score, which may capture different levels or aspects of executive control. Moreover, given the relatively small subgroup size and the number of statistical tests performed, this isolated finding may represent a chance finding (i.e., a type I error). It should therefore be considered preliminary and interpreted with caution until replicated in independent cohorts with larger sample sizes. Overall, our findings support an independent association between self-reported hearing loss and the risk of cognitive decline and dementia. Further exploration of possible additive effects most likely requires a more comprehensive audiological characterization of the population than performed in our study. Overall, we found no evidence for additive effects of self-reported hearing loss and tinnitus, probably due to distinct patterns of brain alterations.

Our findings support the already widely assumed association between hearing loss, cognitive decline, and structural brain changes, without showing evidence for additional associations between tinnitus and the risk of cognitive decline and dementia. On a mechanistic level, the observed mediation effect of WMH volume on the association of self-reported hearing loss and cognitive performance might indicate the presence of cerebrovascular disease in those individuals with. Although our study did not examine hearing rehabilitation, our findings support the growing body of evidence that hearing rehabilitation may reduce the likely contribution of hearing loss to cognitive decline [41]. Participants with self-reported moderate or severe hearing loss in our cohort were older than those reporting little or no hearing loss, reflecting the established age-related increase in hearing impairment [28]. Therefore, we performed additional age-stratified analyses to evaluate whether the observed associations were consistent across different stages of adulthood rather than being solely driven by age differences between self-reported hearing loss categories. These analyses followed the conceptual framework proposed by Livingston and colleagues [3]; however, given the wide age range in our sample with only a limited proportion of participants older than 65 years, we used slightly adapted age cut-offs (midlife: 18–60 years; late life: >60 years) to ensure sufficient sample sizes in each subgroup. The findings of these subgroup analyses suggest that associations between self-reported severe hearing loss and memory performance are already detectable in midlife and persist into late life. In contrast, associations with volumetric brain measures appeared more pronounced in midlife, whereas associations with cortical thickness were mainly observed in late life. One possible explanation is that age-related neurodegenerative processes become increasingly dominant with advancing age; however, this interpretation remains speculative and requires longitudinal investigation. Taken together, these findings underscore the importance of early identification and management of hearing loss from midlife onwards while highlighting the need for longitudinal studies to clarify the mechanisms linking hearing loss, brain structural changes, and cognitive decline, and to determine whether early intervention can reduce the risk of dementia.

This large population-based cohort with comprehensive baseline assessment, including MRI, provided an ideal basis to evaluate associations between self-reported hearing loss and tinnitus in relation to cognitive function and brain health. However, several limitations have to be considered for the interpretation of our results: First, both cognitive tests (wordlist and Stroop test) inherently depend on basic speech perception, which might be affected by hearing loss and tinnitus. Thus, individuals with hearing impairment may perform worse independent of their cognitive function [42]. This limitation likely applies particularly to the wordlist task, whereas visually based tasks such as the Stroop test might be less affected, potentially explaining the differing associations observed. Accordingly, the negative association of self-reported hearing loss with encoding performance might partially reflect difficulties in perceiving and comprehending verbally presented information. However, all participants undergoing cognitive testing were required to show sufficient everyday communications skills. Second, the evaluation of hearing loss and tinnitus relied on self-report, as no audiometric data was available for reference. Although self-reported hearing loss broadly reflects age- and sex-specific distributions observed in audiometric assessments, granular agreement on severity is only moderate [43]. Moreover, self-reported hearing differs from audiometric measures in certain subgroups: it tends to underestimate hearing loss in older adults [44, 45] and varies by sex, age, education, and lifestyle factors [45, 46]. These discrepancies are not limited to single-item assessments, which are commonly used in population-based studies such as ours. Standardised multi-item questionnaires, such as the Hearing Handicap Inventory for Adults and its revised versions, promise a more comprehensive assessment of perceived hearing difficulties than a single-item question. Although these instruments also show good overall agreement with audiometric measures, systematic differences by age, sex, and race remain [47, 48]. Nevertheless, self-reported hearing loss provides an efficient audiologic phenotype in epidemiologic studies and shows reasonable agreement with audiometric testing [45, 49]. Moreover, perceived hearing ability integrates peripheral sensory hearing function with auditory processing, cognitive status, communication abilities, emotional assessment, and societal participation, comparable to the concept of auditory wellness reflecting the WHO framework of healthy function [50]. For the present research question, perceived hearing ability may therefore be particularly informative because it aspires to capture an ecological assessment of communication abilities in everyday listening situations rather than peripheral auditory sensitivity alone. As such, it may be more directly related to downstream outcomes such as social isolation and depression than pure-tone audiometric thresholds [16]. Third, brain imaging was limited to T1-weighted data on brain structure, allowing no conclusions on functional networks, perfusion, or microstructure. Thus, our analyses only allow drawing conclusions on macrostructural brain measures, including brain volume, cortical thickness, and WMH volume. Lastly, the cross-sectional design precludes causal inference, and the directionality and dynamics of these associations require longitudinal investigation.

## Conclusions

This population-based study demonstrated a high prevalence of self-reported hearing loss and tinnitus in adulthood, supported hearing loss—but not tinnitus—as a risk factor for cognitive decline and dementia, and highlighted a potential role of cerebrovascular pathology. These findings underscore the need for further research on longitudinal cohorts, including early hearing screening and timely use of hearing aids.

## Supplementary Information


Supplementary Material 1.


## Data Availability

Data used in this article was obtained from “SHIP – Study of Health in Pomerania”. Requests to access the data set may be sent to “Forschungsverbund Community Medicine” (community-medicine@uni-greifswald.de) with the reference SHIP/2024/10/D.

## Abbreviations

AD: Alzheimer’s Disease
CI: Confidence interval
eTIV: Estimated total intracranial volume
GM: Grey matter
MRI: Magnetic resonance imaging
ROI: Region of interest
SHIP: Study of Health in Pomerania
SSNHL: Sudden sensorineural hearing loss
WM: White matter
WMH: White matter hypointensity
