# Supplementary material for: Association of self-reported hearing loss and tinnitus with brain structure and cognitive function: a population-based cross-sectional analysis from the Study of Health in Pomerania (SHIP)
Source: Alzheimers Res Ther. 2026 Aug 27;18:192. doi: 10.1186/s13195-026-02166-6 (PMC13527976; doi:10.1186/s13195-026-02166-6)
Supplement: Supplementary file 1 [file 13195_2026_2166_MOESM1_ESM.docx]

**Supplementary appendix**

for the article

**Association of self-reported hearing loss and tinnitus with brain structure and cognitive function: a population-based cross-sectional analysis from the Study of Health in Pomerania (SHIP)**

by

Claudia Schwarz, Stefan Frenzel, Till Ittermann, Ulrike Grittner, Silke M Wortha, Tina Brzoska, Hans J Grabe, Agnes Flöel, Friedrich Ihler

## **Contents**

eMethods 1. Study design and participants	3

eMethods 2. Brain imaging outcomes	3

eMethods 3. Statistical analysis	3

eResults 1. Interaction of hearing loss and tinnitus	4

eTable 1. Characteristics of participants included versus excluded from the mediation analyses.	5

eTable 2. Characteristics of study participants stratified by levels of self-reported hearing loss. 6

eTable 3. Cognitive performance measures. 7

eTable 4. Structural brain measures.	8

eTable 5. Association of self-reported moderate and severe hearing loss with cognitive function stratified by midlife and late life. 9

eTable 6. Associations of self-reported severe hearing loss with brain measures stratified by midlife and late life.	10

eTable 7. Mediating role of brain measures on the relationship between self-reported hearing loss and memory performance.	11

eTable 8. Contingency table of self-reported hearing loss and tinnitus.	12

eTable 9. Interactive effects of hearing loss and tinnitus on cognitive performance and brain structure.	13

eFigure 1. Association of hearing loss and tinnitus.	15

eReferences	16

## **eMethods 1. Study design and participants**

SHIP is not focused on a specific disease or organ system, but instead attempts to describe health-related conditions with the widest focus possible. Important medical areas of investigation include cardiovascular diseases, diabetes mellitus, liver and biliary tract diseases, neurological diseases, thyroid diseases, dental diseases, lung diseases, addiction and risk behavior. SHIP consists of independent cohorts. The first two cohorts are named SHIP-START and SHIP-TREND. Those two cohorts finished baseline investigations in the past and already underwent one or more follow-up investigations. A third cohort (SHIP-NEXT) is currently in the process of sampling and baseline investigations; the data is not yet available. All cohorts are sampled from defined parts of mainland Western Pomerania (Vorpommern), a rural area in the northeastern German state of Mecklenburg-Vorpommern. The cohorts do not overlap, as participation in a previous cohort is an exclusion criterion for each cohort. During sampling, adults aged 20–79 were selected from registration data in a randomised, two-stage cluster method stratified by sex and age. While not being an explicit selection criterion, the entire sample is predominantly Caucasian due to the characteristics of the underlying population.

## **eMethods 2. Brain imaging outcomes**

T1-weighted three-dimensional magnetization-prepared rapid acquisition gradient echo (MPRAGE) images were acquired with the following parameters: axial orientation, repetition time = 1,900 ms, echo time = 3.37 ms, flip angle = 15°, slice thickness = 1 mm, and resolution = 1 mm × 1 mm. Further details have been described by Hosten and colleagues (1). Volumetric brain outcomes are expressed in cubic centimeters for easier readability and cortical thickness variables are expressed in millimeters.

## **eMethods 3. Statistical analysis**

To test the association between hearing loss and tinnitus using Fisher’s exact test, both variables were transformed into binary categories. Hearing loss was dichotomised as described for the mediation analysis, and tinnitus was dichotomised by combining grades 0 (“no”) and 1 (“sometimes”) as no substantial tinnitus, and grades 2 (“frequently”) and 3 (“always”) as substantial tinnitus. Associations between hearing loss and tinnitus were also analyzed using the chi-square test, and Pearson residuals were examined to assess cell-specific deviations and identify the cells contributing most strongly to the chi-square statistic.

## **eResults 1. Interaction of hearing loss and tinnitus**

We observed an association between the occurrence of hearing loss and tinnitus χ2(9, N=3,923)=555.4, p<0.001). The most contributing cells to the Chi-square are “always tinnitus” and “self-reported moderate hearing loss” (27.1%) as well as “always tinnitus” and “self-reported severe hearing loss” (22.6%). These cells contribute about 50% to the total Chi-square score and thus account for half of the difference between expected and observed values.

Moreover, participants with hearing loss but no tinnitus showed worse cognitive function in all sub scores of the wordlist (all p’s<0.003) compared to participants with neither hearing loss nor tinnitus. Regarding brain structure, we observed that participants with hearing loss but no tinnitus showed smaller mean cortical thickness (*B* -0.026 [95% CI -0.043 to -0.009]; p=0.003) and superior temporal cortical thickness (*B* -0.048 [95% CI -0.073 to -0.023]; p<0.001) as well as smaller hippocampal volume (*B* -139.2 [95% CI -247.6 to -30.8]; p=0.012) and greater WMH volume (*B* 668.2 [95% CI 253.4 to 1083.1]; p=0.002) compared to participants with neither hearing loss nor tinnitus.

As we did not find any additive effect of hearing loss and tinnitus or associations of tinnitus alone with cognitive performance, our preconceived hypotheses were not applicable and therefore no mediation models were calculated in this case.

## **eTable 1. Characteristics of participants included versus excluded from the mediation analyses.**

|  | **Included (N=2,050)** | **Excluded (N=2,370)** |
| --- | --- | --- |
| Females [N] (%) | 1,060 (52) | 1,215 (51) |
| Age [years] | 51 (14), 21-82 | 52 (17), 20-84 |
| Education [years] | 13 (2), 0-17 | 12 (3), 0-17 |
| Hearing loss [N] (%) |  |  |
| no | 1,762 (86) | 1941 (82) |
| yes | 288 (14) | 409 (17) |

Data are given as mean (standard deviation) and range or as number (%), for participants included in the mediation analyses and those excluded due to incomplete data, respectively. Missing data for education years (N=70) and hearing loss (N=20) are reported for the excluded group.

## **eTable 2. Characteristics of study participants stratified by levels of self-reported hearing loss.**

|  | **Hearing loss (N = 4,400)** |
| --- | --- |

| **Characteristic** | **Not at all**  (N=2,945) | **Rarely**  (N=758) | **Moderately**  (N=541) | **Severely**  (N=156) |
| --- | --- | --- | --- | --- |
| Females [N] (%) | 1,643 (55.8) | 348 (45.9) | 199 (36.8) | 74 (47.4) |
| Age [years] | 46 (15), 20-83 | 55 (14), 23-82 | 61 (14), 22-84 | 64 (14), 27-82 |
| Education [years] | 12 (3), 0-17 | 12 (3), 0-17 | 12 (3), 0-17 | 11 (3), 0-17 |

Data are given as mean (standard deviation) and range or number (%). Missing data for education years are reported for the hearing loss categories not at all (N=38), rarely (N=7), moderately (N=15), and severely (N=4).

## **eTable 3. Cognitive performance measures.**

| **Parameter** | **Total sample (N = 4,375)** |
| --- | --- |
| **Nuremberg Gerontopsychological Inventory, wordlist** |  |
| Wordlist, total [score] | 10.9 (2.7), -1-16 |
| Wordlist, immediate recall [score] | 5.2 (1.4), 0-8 |
| Wordlist, delayed recognition [score] | 5.7 (1.7), -3-8 |
| **Stroop Colour-Word Test (N = 4,025)** |  |
| Stroop interference score [s] | 20.3 (13.0), -10-331 |

Data are given as mean (standard deviation) and range. Missing data for Stroop test (N=350) is reported. Abbreviations: s, seconds.

## **eTable 4. Structural brain measures.**

| **Parameter** | **Total sample (N = 2,144)** |
| --- | --- |
| **Global measures** |  |
| total GM volume [cm^3^] | 633.7 (62.7) |
| cortical GM volume [cm^3^] | 463.3 (50.2) |
| subcortical GM volume [cm^3^] | 55.1 (5.3) |
| cerebellar GM volume [cm^3^] | 114.5 (12.1) |
| cerebral WM volume [cm^3^] | 484.4 (60.0) |
| cerebellar WM volume [cm^3^] | 26.7 (4.1) |
| WMH volume [cm^3^] | 1.4 (2.5) |
| mean cortical thickness [mm] | 2.4 (0.1) |
| **Regional measures** |  |
| brain stem volume [cm^3^] | 21.8 (2.4) |
| thalamus volume [cm^3^] | 14.6 (1.7) |
| hippocampus volume [cm^3^] | 7.9 (0.8) |
| superior temporal gyrus volume [cm^3^] | 24.0 (3.0) |
| superior temporal gyrus cortical thickness [mm] | 2.7 (0.2) |

Data are given as mean (standard deviation) and range. Abbreviations: GM, grey matter; WM, white matter; WMH, white matter hypointensity.

## **eTable 5. Association of self-reported moderate and severe hearing loss with cognitive function stratified by midlife and late life.**

|  | **Midlife (18–65 years)** | | | | **Late life (>65 years)** | | | |
| --- | --- | --- | --- | --- | --- | --- | --- | --- |
|  | **Estimate** | **95% CI** | **t-value** | **p-value** | **Estimate** | **95% CI** | **t-value** | **p-value** |
| **Self-reported moderate hearing loss** |  |  |  |  |  |  |  |  |
| Wordlist, total [score] | -0.36 | -0.68 to -0.04 | -2.23 | 0.026 | -0.27 | -0.68 to 0.14 | -1.29 | 0.200 |
| Wordlist, immediate recall [score] | -0.21 | -0.37 to -0.05 | -2.54 | 0.011 | -0.31 | -0.40 to -0.01 | -2.11 | 0.035 |
| Wordlist, delayed recognition [score] | -0.15 | -0.36 to 0.06 | -1.40 | 0.161 | -0.06 | -0.66 to 0.04 | -0.42 | 0.673 |
| Stroop interference score [s] | -1.06 | -2.20 to 0.07 | -1.84 | 0.066 | 0.22 | -2.09 to 2.54 | 0.19 | 0.717 |
| **Self-reported severe hearing loss** |  |  |  |  |  |  |  |  |
| Wordlist, total [score] | -0.65 | -1.30 to -0.001 | -1.96 | 0.050 | -1.02 | -1.65 to -0.40 | -3.21 | 0.001 |
| Wordlist, immediate recall [score] | -0.41 | -0.74 to -0.08 | -2.43 | 0.015 | -0.69 | -0.98 to -0.39 | -4.57 | <0.001 |
| Wordlist, delayed recognition [score] | -0.24 | -0.67 to 0.20 | -1.08 | 0.281 | -0.33 | -1.05 to -0.02 | -1.51 | 0.132 |
| Stroop interference score [s] | -1.25 | -3.62 to 1.12 | -1.04 | 0.300 | 2.89 | -0.78 to 6.56 | 1.54 | 0.123 |

Linear models on the association between self-reported hearing loss and cognitive performance (wordlist, N=3,978; Stroop test, N=3,653) stratified by midlife (wordlist, n=2,776; Stroop test, n=2,570) and late life (wordlist, n=1,202; Stroop test, n=1,083), adjusted for age, sex, education, metabolic syndrome, depression, physical activity, smoking, alcohol consumption, social isolation, and vision. Abbreviations: CI, confidence interval; s, seconds.

## **eTable 6. Associations of self-reported severe hearing loss with brain measures stratified by midlife and late life.**

|  | **Midlife (18–65 years)** | | | | **Late life (>65 years)** | | | |
| --- | --- | --- | --- | --- | --- | --- | --- | --- |
|  | **Estimate** | **95% CI** | **t-value** | **p-value** | **Estimate** | **95% CI** | **t-value** | **p-value** |
| **Brain structure** |  |  |  |  |  |  |  |  |
| total GM volume [cm^3^] | -11.31 | -22.51 to -0.11 | -1.98 | 0.048 | -4.98 | -15.29 to 5.32 | -0.95 | 0.343 |
| cortical GM volume [cm^3^] | -10.80 | -20.70 to -0.89 | -2.14 | 0.033 | -3.75 | -13.05 to 5.56 | -0.79 | 0.429 |
| subcortical GM volume [cm^3^] | -1.89 | -3.21 to -0.57 | -2.81 | 0.005 | -0.50 | -1.57 to 0.58 | -0.91 | 0.364 |
| cerebral WM volume [cm^3^] | -20.40 | -34.12 to -6.69 | -2.92 | 0.004 | 2.18 | -10.25 to 14.61 | 0.34 | 0.731 |
| cerebellar GM volume [cm^3^] | 1.19 | -2.87 to 5.24 | 0.58 | 0.565 | 0.75 | -4.16 to 2.67 | 0.43 | 0.668 |
| cerebellar WM volume [cm^3^] | -0.68 | -2.24 to 0.89 | -0.85 | 0.398 | 0.76 | 0.56 to 2.09 | 1.13 | 0.259 |
| WMH volume [cm^3^] | 0.29 | -0.05 to 0.63 | 1.68 | 0.093 | -0.03 | -1.42 to 1.48 | 0.04 | 0.969 |
| mean cortical thickness [mm] | -0.01 | -0.05 to 0.03 | -0.51 | 0.613 | -0.05 | -0.09 to -0.01 | -2.64 | 0.008 |
| brain stem volume [cm^3^] | -0.27 | -1.06 to 0.51 | -0.69 | 0.491 | -0.57 | -1.22 to -0.08 | -1.72 | 0.087 |
| thalamus volume [cm^3^] | -0.66 | -1.16 to -0.16 | -2.58 | 0.010 | -0.08 | -0.45 to 0.30 | -0.40 | 0.689 |
| hippocampus volume [cm^3^] | -0.22 | -0.48 to 0.05 | -1.62 | 0.106 | -0.15 | -0.37 to 0.08 | -1.27 | 0.205 |
| superior temporal gyrus volume [cm^3^] | -0.73 | -1.66 to 0.20 | -1.55 | 0.122 | -0.03 | -1.06 to 0.43 | -0.84 | 0.403 |
| superior temporal gyrus cortical thickness [mm] | -0.01 | -0.07 to 0.05 | -0.23 | 0.816 | -0.08 | -0.14 to -0.03 | -2.86 | 0.004 |

Linear models on the association between self-reported hearing loss and brain measures (N=2,065) stratified by midlife (n=1,492) and late life (n=573), adjusted for age, sex, education, metabolic syndrome, depression, physical activity, smoking, alcohol consumption, social isolation, and vision. Models including volume measures as the dependent variable were further adjusted for estimated total intracranial volume. Abbreviations: CI, confidence interval; GM, grey matter; WM, white matter; WMH, white matter hypointensity.

## **eTable 7. Mediating role of brain measures on the relationship between self-reported hearing loss and memory performance.**

|  | **Proportion mediated** | **Indirect effect** | | |
| --- | --- | --- | --- | --- |
|  | **Estimate** | **Estimate** | **95% CI** | **p-value** |
| cerebral WM volume | 0.013 | -0.003 | -0.012 to 0.002 | 0.356 |
| subcortical GM volume | 0.016 | -0.004 | -0.013 to 0.002 | 0.241 |
| brain stem volume | 0.012 | -0.003 | -0.012 to 0.003 | 0.424 |
| thalamus volume | 0.017 | -0.004 | -0.013 to 0.002 | 0.239 |
| hippocampus volume | 0.028 | -0.006 | -0.018 to 0.001 | 0.131 |
| mean cortical thickness | 0.008 | -0.002 | -0.010 to 0.004 | 0.570 |
| superior temporal gyrus cortical thickness | 0.025 | -0.006 | -0.018 to 0.003 | 0.206 |

Mediation models on the mediating effect of structural brain measures on the relationship between hearing loss and immediate recall performance (N=2,050), adjusted for age, sex, education years, metabolic syndrome, depression, physical activity, smoking, alcohol consumption, social isolation, and vision. The proportion mediated by the respective brain measures as well as the partially standardised estimates, 95% confidence interval (CI) and p-values for the indirect effect are reported, adjusted for age, sex, education, metabolic syndrome, depression, physical activity, smoking, alcohol consumption, social isolation, and vision. Models including volume measures as the mediator variable were further adjusted for estimated total intracranial volume. Abbreviations: GM, grey matter; WM, white matter.

## **eTable 8. Contingency table of self-reported hearing loss and tinnitus.**

|  |  | **Tinnitus** | | | |
| --- | --- | --- | --- | --- | --- |
|  |  | 1 - no | 1 - sometimes | 2 - frequently | 3 - always |
| **Self-reported hearing loss** | 1 - not at all | 2,273 (57.9) | 235 (6.0) | 60 (1.5) | 78 (2.0) |
|  | 2 - rarely | 489 (12.5) | 104 (2.7) | 36 (0.9) | 52 (1.3) |
|  | 3 - moderately | 237 (6.0) | 75 (1.9) | 51 (1.3) | 103 (2.6) |
|  | 4 - severely | 53 (1.4) | 21 (0.5) | 13 (0.3) | 43 (1.1) |

A total of 3,923 individuals were recorded with data on self-reported hearing loss and tinnitus, of whom 498 individuals reported that they experienced both hearing loss and tinnitus. Number and percentage are reported for each cell.

## **eTable 9. Interactive effects of self-reported hearing loss and tinnitus on cognitive performance and brain structure.**

|  | **Estimate** | **95% CI** | **t-value** | **p-value** |
| --- | --- | --- | --- | --- |
| **Cognition** |  |  |  |  |
| Wordlist, total [score] | -0.19 | -0.48 to 0.11 | -1.24 | 0.214 |
| Wordlist, immediate recall [score] | -0.22 | -0.37 to -0.08 | -2.98 | 0.003 |
| Wordlist, delayed recognition [score] | 0.04 | -0.16 to 0.24 | 0.35 | 0.726 |
| Stroop interference score [s] | -1.42 | -2.76 to -0.08 | -2.08 | 0.037 |
| **Brain structure** |  |  |  |  |
| total GM volume [cm^3^] | 1.50 | -2.98 to 5.99 | 0.66 | 0.511 |
| cortical GM volume [cm^3^] | 0.45 | -3.53 to 4.42 | 0.22 | 0.826 |
| subcortical GM volume [cm^3^] | 0.38 | -0.47 to 0.54 | 0.15 | 0.883 |
| cerebral WM volume [cm^3^] | -1.53 | -1.53 to 2.81 | -0.54 | 0.586 |
| cerebellar GM volume [cm^3^] | 1.02 | -0.53 to 2.57 | 1.29 | 0.199 |
| cerebellar WM volume [cm^3^] | 0.22 | -0.38 to 0.82 | 0.72 | 0.471 |
| WMH volume [cm^3^] | 0.13 | -0.27 to 0.53 | 0.62 | 0.534 |
| mean cortical thickness[mm] | 0.01 | -0.01 to 0.03 | 1.33 | 0.185 |
| brain stem volume [cm^3^] | 0.05 | -0.25 to 0.35 | 0.33 | 0.738 |
| thalamus volume [cm^3^] | -0.02 | -0.21 to 0.17 | -0.20 | 0.845 |
| hippocampus volume [cm^3^] | -0.01 | -0.11 to 0.10 | -0.13 | 0.899 |
| superior temporal gyrus volume [cm^3^] | 0.06 | -0.29 to 0.42 | 0.35 | 0.724 |
| superior temporal gyrus cortical thickness [mm] | 0.01 | -0.02 to 0.03 | 0.38 | 0.708 |

Linear models on the interaction of self-reported hearing loss and tinnitus and its association with cognitive performance (wordlist: N=3,750; Stroop test: N=3,430) and brain structure (N=2,044), adjusted for age, sex, education, metabolic syndrome, depression, physical activity, smoking, alcohol consumption, social isolation, and vision. Models including volume measures as the dependent variable were further adjusted for estimated total intracranial volume. Abbreviations: CI, confidence interval; GM, grey matter; s, seconds; WM, white matter; WMH, white matter hypointensity.

## **eFigure 1. Association of self-reported hearing loss and tinnitus.**

**Tinnitus**

*No*

*Always*

*Frequently*

*Sometimes*

*Not at all*

*Rarely*

**Hearing loss**

*Moderately*

*Severely*

Correlation plot with Pearson residuals for the occurrence of self-reported hearing loss and tinnitus (N=3,923). The size of the circle in each cell is proportional to the amount of the cell contribution. Positive residuals (= positive associations) are in blue and negative residuals (= negative associations or no associations) are indicated by red colour. The most contributing cells to the Chi-square are always tinnitus and self-reported moderate hearing loss (27.1%) as well as always tinnitus and self-reported severe hearing loss (22.6%).

## **eReferences**

1.	Hosten N, Bülow R, Völzke H, Domin M, Schmidt CO, Teumer A, et al. SHIP-MR and Radiology: 12 Years of Whole-Body Magnetic Resonance Imaging in a Single Center. Healthcare (Basel, Switzerland). 2021;10(1).
